# A meta-analysis of systematic reviews and meta-analyses to evaluate the psychological consequences of COVID-19

**DOI:** 10.1186/s40359-023-01313-0

**Published:** 2023-09-18

**Authors:** Massoud Sokouti, Ali Reza Shafiee-Kandjani, Mohsen Sokouti, Babak Sokouti

**Affiliations:** 1 Research Center of Psychiatry and Behavioral Sciences, Tabriz University of Medical Sciences, Tabriz, Iran; 2 Department of Cardiothoracic Surgery, Tabriz University of Medical Sciences, Tabriz, Iran; 3 Biotechnology Research Center, Tabriz University of Medical Sciences, Tabriz, Iran

**Keywords:** Meta-analysis of meta-analyses, COVID-19, Psychological symptoms

## Abstract

**Background:**

Several meta-analysis studies have been reported in the literature on the incidence of psychopathological conditions resulting from the COVID-19 pandemic. This investigation aims to compile and analyze the findings of previously published meta-analysis research, as shown by the present meta-analysis of previous meta-analysis studies.

**Methods:**

The PubMed and Scopus databases were searched from 1 January 2019 to 30 May 2022. The procedure was carried out according to the PRISMA flow chart and the qualities of the identified studies were analyzed using AMSTAR 2. Heterogeneities and risk of bias were assessed using the Meta-MUMS tool. The corresponding results, forest and funnel plots of the psychological consequences of COVID-19 were synthesized.

**Results:**

Eleven meta-analysis studies were included. Random-effects meta-analysis of anxiety and depression showed (ER = 0.318 *p-value* < 0.001, ER = 0.295 *p-value* < 0.001) high heterogeneities (I^2^ = 99.70%, I^2^ = 99.75) between studies. Random-effects meta-analyses of sleep difficulties and insomnia were shown (ER = 0.347 *p-value* < 0.001, ER = 0.265, *p-value* < 0.001) along with heterogeneities (I^2^ = 99.89, I^2^ = 99.64). According to the random meta-analysis of post-traumatic stress syndrome (PTSS) and post-traumatic stress disorder (PTSD) (ER = 0.246, *p-value* = 0.001, ER = 0.223 *p-value* < 0.001) with heterogeneities (I^2^ = 99.75, I^2^ = 99.17). Random-effects meta-analyses of somatic and fear symptoms have been shown (ER = 0.16 *p-value* < 0.001, ER = 0.41, *p-value* = 0.089) with high heterogeneities (I^2^ = 99.62, I^2^ = 98.63). Random-effects meta-analysis of obsessive–compulsive symptoms and distress (ER = 0.297 *p-value* = 0.103; ER = 0.428, *p-value* = 0.013*)* with high heterogeneity, as I^2^ = 99.38%. Subgroup analysis of all symptoms and Egger's tests for detecting publication bias were also assessed.

**Conclusion:**

The data from the current meta-analysis showed different psychological disorders of COVID-19 during the pandemic. Clinicians should be aware of the prevalence with which COVID-19-infected patients experience emotional distress, anxiety, fatigue, and PTSD. About half of the included systematic reviews (SRs)/meta-analyses (MAs) suffered from poorer methodological quality and increased risk of bias, reducing confidence in the findings. There must be more SRs/MAs and high-quality clinical trials conducted to confirm these findings.

**Supplementary Information:**

The online version contains supplementary material available at 10.1186/s40359-023-01313-0.

## Introduction

One of the most serious health problems that threatens the world population is COVID-19 infection. According to the weekly WHO report, as of 31 May 2023, approximately 767 million confirmed cases and near seven million deaths had been reported worldwide (https://covid19.who.int/). The world's policy makers need to pay more attention to how the COVID-19 pandemic could affect people's mental and psychological well-being [1]. In response to this pandemic, measures were taken to contain the virus in most of the inhabited regions. The COVID-19 pandemic, also known as SARS-CoV-2, leads to manifestations comparable to severe acute respiratory syndrome (SARS).

The current research is based on the hypothesis of a stress diathesis model in global health priority (WHO). And, the conceptual framework of is based on psychiatric diagnosis and psychological symptoms.

Psychiatric disorders are obsessive compulsive symptoms (OCS), posttraumatic stress disorders (PTSD), phobia, sleep problems, and post-traumatic stress syndrome (PTSS) and psychological symptoms such as anxiety, depression, distress, somatic symptoms insomnia, sleep problems, fear, and other psychiatric symptoms among healthcare personnel and the general public people. Several studies also reported mental illness due to COVID-19 [2–14].

The obsessive–compulsive disorder (OCD) cycle has four basic parts: obsessions, anxiety, compulsions, and temporary relief and has a vicious cycle. A person with anxiety is worried or afraid, and a person with depression is one who loses interest and enjoyment in things because of low mood or a lack of interest. A sleep disorder known as insomnia affects the brain and affects daily activities. Insomnia can make you sleepy during the day, so you cannot accomplish your daily tasks.

A stress response occurs when an environment or internal disturbance triggers an adaptive response. An individual who is stressed excessively and for a prolonged period of time suffers from distress. The possibility of distress that arises from an experiment can be distinguished from the potential welfare benefit of such an experiment [15].

It has been reported that COVID-19 infection results in cytokines being secreted and activation of the kynurenine pathway promoting tryptophan metabolism. Psychological disorders including schizophrenia, bipolar disorder, depression, and suicide are associated with disruptions of the limbic circuits caused by these chemicals [16].

Infection of the central nervous system (CNS) by SARS-COV-2 has caused meningitis, encephalitis, and detectable RNA in the cerebrospinal fluid (CSF) of patients with COVID-19 [16, 17].

There may also be an effect of inflammation on the development of dementia-like symptoms in association with COVID-19 infection. COVID-19 can initiate inflammation and cause manic symptoms in the acute stage of a disease by producing the interleukins IL-6, IL-10, and plasma C-reactive protein (CRP) [18, 19].

The clinical implications of COVID-19 infections include the risk of infection, loss of loved ones, burden of disease (BOD), dally, quality of life (QOL), feelings of isolation and loneliness, exhaustion of healthcare personnel on a physical and emotional level, permanent fear Obsession and widespread loss in high-, low-, and middle-income countries are all related to a variety of mental health issues [2, 20, 21]. These implications of COVID-19 infection can cause significant problems in the world.

Millions of people around the globe may have been affected by psychiatric disorders and that figure may continue to grow as the number of people infected with COVID-19 increases.

By examining the psychological consequences of diverse populations throughout the countries, a theoretical framework susceptibility developed for identifying stress diathesis in high-risk people, it can be hypothesized that vulnerable individuals will have worse outcomes after a Covid-19 infection,

Some meta-analyses have shown that COVID-19-infected patients may have varied psychological presentations [4]. The population affected by COVID-19 was shown to have a somewhat high incidence of psychiatric disorders and psychological symptoms according to systematic reviews and meta-analyses [22]. And our aim is to evaluate the occurrence rates of patients with psychological and mental conditions caused by COVID-19 and to determine the sample sizes and event rates (ER) of these patients from available meta-analysis studies. This review is based on general consideration, literature review, paradox gaps of knowledge, and clinical implications. It is also intended to examine the pooled population prevalence of mental health issues. Due to this, we decided to undertake a meta-analysis of meta-analyses to evaluate the most prevalent psychiatric disorders due to the recent COVID-19 pandemic among healthcare personnel, the general population, and patients with preexisting problems.

## Materials and methods

The current meta-analysis of meta-analyses was conducted using a search protocol from 1 January 2019 to 30 May 2022 to detect the psychological effect of post-COVID-19 infection. PRISMA 2020 has been designed for systematic review of studies that evaluate the disorders of post-COVID-19 infection disorders. We extracted the event rates of depression, anxiety, distress, PTSS, PTSD, somatic complaints, fear, obsessive–compulsive symptoms, insomnia, and sleep problems originating from COVID-19 infection from the electronic databases of PubMed and Scopus. The search strategy for PubMed was ((COVID-19[Title]) OR(Sars-Cov-2[Title])) AND (meta-analysis[Title]) AND (psychiatry [Title] OR psychological [Title] OR psychiatric [Title]) and for Scopus it was (TITLE ( covid-19) OR TITLE ( sars-cov-2)) AND TITLE ( meta-analysis) AND TITLE ( psy*). Two reviewers independently read the titles and abstracts of the retrieved publications and decided whether they were appropriate for inclusion in the meta-analysis. Checklist items are used to report the results of systematic reviews to evaluate the event rates and prognosis of psychopathological disorders of COVID-19 infection. The PRISMA 2020 is intended for the synthesis of qualitative data according to the protocol. Statistical data synthesis of studies through performing meta-analysis for combining *p-value*s, observed and effect estimates were done. Outcome measurements collected for the participants in each study, such as mortality and quality of life, were determined. Duplicate articles with similar reports and types of studies are excluded (Supplementary Material) [23].

The inclusion criteria were obtained from the union of included outcome within the systematic review and meta-analysis studies. Hence, those studies were considered for inclusion in the investigation if they met the following criteria: comprised of a meta-analytical study measuring symptoms of depression, anxiety, or sleep disorders; peer-reviewed articles published in the English language; patients gave adequate information to compute prevalence / and event rates with sample sizes of the condition; and somatic and fear symptoms, insomnia, distress, and obsessive–compulsive disorders were also evaluated. Articles with systematic reviews, inadequate data, or irrelevant meta-analyses were excluded. According to the included studies, psychological symptoms of patients with COVID-19 infection were obtained by questionnaires.

### Article quality assessment

We used the AMSTAR 2 checklist and its sixteen domains scored as "yes," "no," "partial yes," and "no meta-analysis." Six systematic reviews/meta-analyses (SRs/MAs) were found to have critically poor quality based on AMSTAR 2 because each had several unmet crucial domains. Five SRs/MAs had one noncritical weakness, two had one major defect with or without non-critical flaws, and four articles had more than one major defect with or without non-critical faults, respectively, making their overall quality high, low, and critical low (Table 1). The terms “high,” "moderate," "low," and "critically low" are used to describe overall quality [24]. Reviews that were rated low or critically low were not removed from the study as they might include required information that met the inclusion criteria.

**Table 1:** Methodological quality assessment by AMSTAR2

Systematic Reviews	Item 1	Item 2	Item 3	Item 4	Item 5	Item 6	Item 7	Item 8	Item 10	Item 11	Item 12	Item 13	Item 14	Item 15	Item 16	Quality
Dong F 2021 [25]	Y	Y	Y	Y	Y	Y	Y	Y	Y	Y	N	N	Y	Y	Y	Low
Singh 2021 [26]	Y	Y	Y	Y	Y	Y	Y	Y	N	Y	N	N	Y	N	N	Critical Low
Sun 2021 [27]	Y	Y	Y	Y	Y	Y	Y	Y	Y	Y	Y	N	N	Y	Y	Low
Ching 2021 [28]	Y	Y	Y	Y	Y	Y	Y	Y	Y	Y	Y	Y	Y	Y	Y	High
Alimoradi 2021 [29]	Y	Y	Y	Y	Y	Y	Y	Y	Y	Y	Y	Y	Y	Y	Y	High
Li Wei 2021 [30]	Y	Y	Y	Y	Y	Y	Y	Y	Y	Y	N	N	Y	N	Y	Critical Low
Da silva 2021 [31]	Y	Y	Y	Y	Y	Y	Y	Y	Y	Y	Y	N	N	N	Y	Critical Low
Khraisat 2022 [32]	Y	Y	Y	Y	Y	Y	Y	Y	Y	Y	N	Y	Y	Y	Y	High
Cenat 2021 [20]	Y	Y	Y	Y	Y	Y	Y	Y	Y	Y	Y	Y	N	Y	Y	High
Batra 2020 [33]	Y	Y	Y	Y	Y	Y	Y	Y	Y	Y	Y	Y	Y	Y	Y	High
Luo 2020 [34]	Y	Y	Y	Y	Y	Y	Y	Y	Y	Y	N	N	Y	N	Y	Critical Low

Item 1 Component PICO; Item 2 = Review Protocol; Item 3 = Explanation for study design; Item 4 = Cumulative research strategy; Item 5 = study selection in duplicate; Item 6 = Data extraction in duplicate; Item 7 = List of excluded studies and justify the exclusions; Item 8 = Study characteristics?; Item 9 = satisfactory for assessing the risk of bias; Item 10 = Sources of funding; Item 11 = Appropriate methods; Item 12 = Access potential impact of RoB on the results; Item 13 = Account RoB when heterogeneity discussing; Item 14 = Satisfactory explanation and discussion of any heterogeneity; Item 15 = Publication bias assessed and discussed; Item 16 = Potential sources of conflict of interests

An Excel spreadsheet was used to gather the results of the data retrieved blindly by two reviewers. Both the research findings and features of investigations, such as the number of studies, the event rates, and the sample sizes, were subjected to data collection (heterogeneity of event rates and *I*^2^). Forrest and funnel plots and determining publication bias using Egger tests were also performed [35].

### Meta-analysis

We used the Meta-MUMS tool to carry out the studies [36–44]. The prevalence rate was equivalent to the event rate. The I^2^ statistic, used to measure inconsistency, was used to analyze the degree of variation across studies (heterogeneity) [45]. Low levels of heterogeneity were defined as I^2^ = 25–49%, moderate levels as I^2^ = 50–74%, and high levels as I^2^ = 75–100% [46, 47]. In each of the analyses, a model with random-effects was used. Pooled random-effects analysis consisted of data from a minimum of three separate investigations to provide sufficient statistical power [48]. We compared anxiety, depression, sleep problems, insomnia, distress, and post-traumatic stress disorder (PTSD) across continents using subgroup data analysis and meta-regression [35, 49]. To compare different subgroups, Cochran's Q and degrees of freedom were presented as Q (df) [50, 51]. For all comparisons between subgroups, a *p-value* of 0.05 was considered significant. When funnel plots had an asymmetrical appearance, Egger's test of effect size versus its standard error or trim and fill procedures were carried out. This was done so that publication bias could be identified [52, 53].

Heterogeneity tests and meta-regression [35, 54–56]. Trim and fill operations [57] were performed for anxiety, depression, stress, sleep problems, insomnia, distress, PTSD, and somatic symptoms.

## Results

**Fig. 1:**
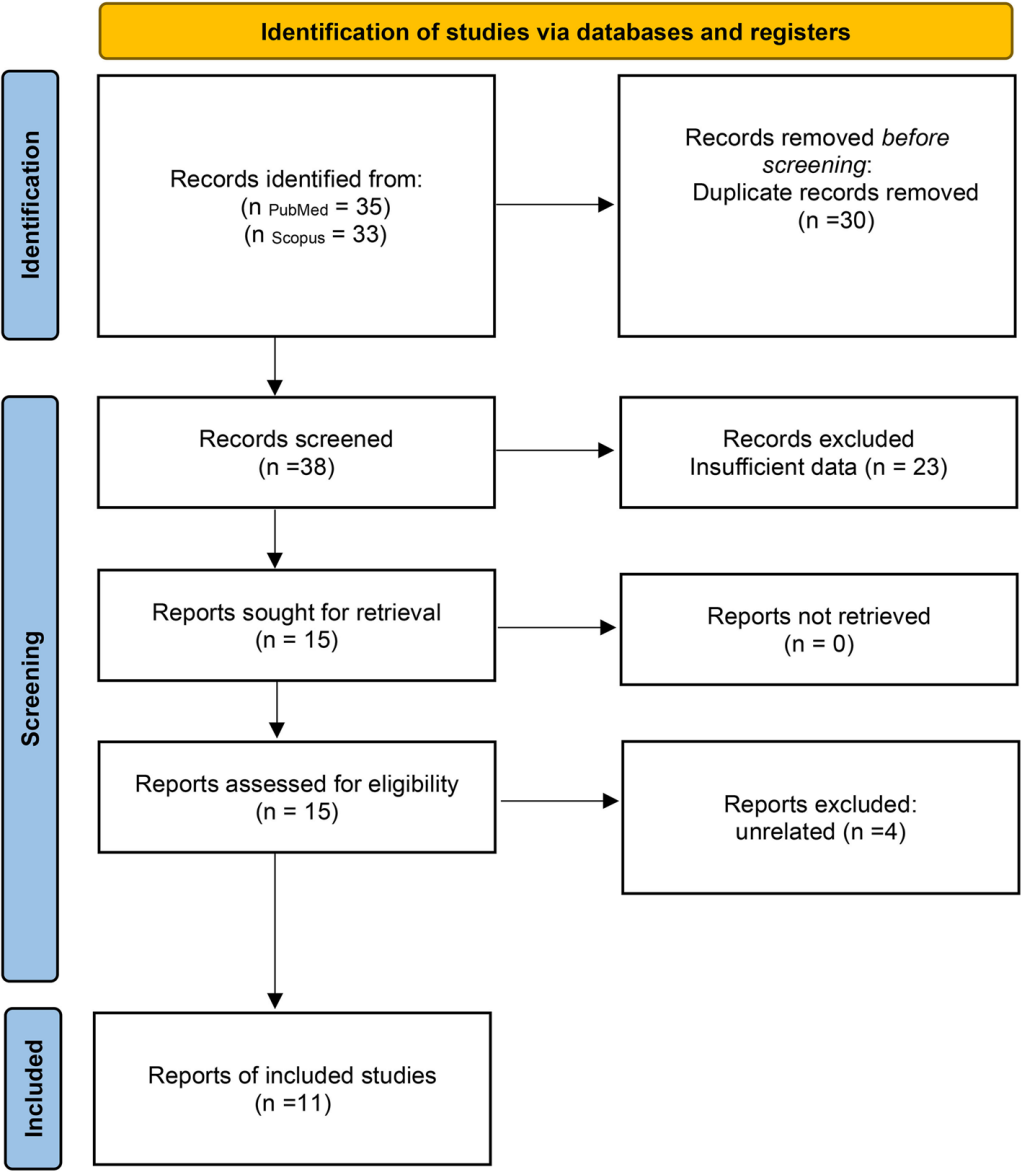
PRISMA flowchart of the systematic review procedure

Of the studies that passed the first title/abstract filter, 11 meta-analyses were selected, with a combined sample size of 3,502,427 patients (Fig. 1). Furthermore, a total of eleven meta-analyses were included [20, 25–29, 31, 33, 34, 58, 59]. Four meta-analysis studies were excluded from unrelated articles [60–63]. Total event rates of psychiatric disorders due to COVID-19 included 618,145 patients with anxiety in 306 articles, 593,894 patients with depression in 259 articles, 28,148 patients with PTSS in 7 articles, 37,353 patients with PTSD in 35 articles, 2,029,837 patients with sleep problems in 201 articles, 15,639 patients with somatic symptoms in 9 articles, 22,956 patients with fear symptoms in 7 articles, 3721 patients with obsessive–compulsive disorder included in 5 articles, 61,720 patients with insomnia in 25 articles, 91,924 patients with distress in 22 articles. The risk of bias in studies were performed in all psychologic behaviors of post-covid infection patients. Summary statistics and synthesis of the studies are obtained on structured tables and plots and determining the risk of bias among the contributing studies.

The PRISMA flow chart (Fig. 1) shows the type of selection of the current study. Four out of 11 studies were conducted in China, while the remaining were conducted in the United States, India, Brazil, Iran, Malaysia, United Arab Emirates and Canada.

Subgroup analysis was not performed on obsessive–compulsive symptoms, fear and somatic symptoms, and PTSS due to lack of data, while serving on other psychological disorders. For detecting publication bias, the Egger’s test was performed. Tables 2 and 3 show the listed data from the meta-analysis of subgroup and heterogeneity.

**Table 2:** Subgroup random meta-analysis of anxiety, depression, sleep problem, insomnia, distress, post-traumatic stress disease (PTSD) based on continents in 11 studies

**Anxiety**	Continent	*ER* = *0.318*	*LL* = *0.294*	*UL* = *0.345*	*P* < 0.001
		*ER*_Am_ = 0.259	*LL* = 0.163	*UL* = 0.385	*P* < 0.001
		*ER*_AS_ = 0.323	*LL* = 0.295	*UL* = 0.351	*P* < 0.001
		*ER*_EUR_ = 0.300	*LL* = 0.210	*UL* = 0.400	*P* < 0.001
		*ER*_*Afr*_ = *0.47*	LL = 0.100	UL = 0.876	P = 0.910
		*ER*_sum_ = 0.318	*LL* = 0.294	*UL* = 0.345	*P* < 0.001
**Depression**	Continent	*ER* = *0.295*	*LL* = *0.270*	*UL* = *0.322*	*P* < 0.001
		*ER*_Am_ = 0.283	*LL* = 0.173	*UL* = 0.426	P = 0.004
		*ER*_AS_ = 0.300	*LL* = 0.273	*UL* = 0.329	*P* < 0.001
		*ER*_EUR_ = 0.239	*LL* = 0.166	*UL* = 0.331	*P* < 0.001
		*ER *_*Afr*_ = 0.56	*LL* = 0.148	*UL* = 0.904	P = 0.813
		*ER*sum = 0.295	*LL* = 0.270	*UL* = 0.322	*P* < 0.001
**Sleep problem**	Continent	*ER* = *0.347*	*LL* = *0.318*	*UL* = *0.376*	*P* < 0.001
		*ER*_Am_ = 0.536	*LL* = 0.426	*UL* = 0.643	P = 0.523
		*ER*_As_ = 0.288	*LL* = 0.258	*UL* = 0.321	*P* < 0.001
		*ER*_EU_ = 0.451	*LL* = 0.386	*UL* = 0.517	P = 0.145
		ER_Afr_ = 0.489	LL = 0.34	UL = 0.64	P = 0.89
		*ER*_*sum*_ = 0.347	*LL* = 0.329	*UL* = 0.375	*P* < 0.001
**Insomnia**	continent	*ER* = 0.265	*LL* = 0.205	*UL* = 0.337	*P* < 0.001
		*ER*_*asi*_ = 0.286	*LL* = 0.220	*UL* = 0.362	*P* < 0.001
		*ER*_*EUR*_ = 0.177	*LL* = 0.088	*UL* = 0.324	*P* < 0.001
		ER_sum_ = 0.266	LL = 0.208	UL = 0.333	*P* < 0.001
**Distress**	Continent	*ER* = *0.428*	*LL* = 0.372	*UL* = 0.485	P = 0.016
		*ER*_Ame_ = 0.602	LL = 0.336	Ul = 0.819	P = 0.460
		*ER*_ASI_ = 0.454	*LL* = 0.385	*UL* = 0.524	P = 0.199
		*ER*_EUR_ = 0.332	*LL* = 0.238	*UL* = 0.441	P = 0.003
		*ER*_*sum*_ = 0.428	*LL* = 0.372	*UL* = 0.487	P = 0.016
**PTSD**	Continent	*ER* = *0.223*	*LL* = *0.169*	*UL* = *0.290*	*P* < *0.001*
		*ER*_*Asi*_ = *0.257*	*LL* = *0.166*	*UL* = *0.375*	*P* < *0.001*
		*ER*_*EUR*_ = 0.191	*LL* = 0.106	*UL* = *0.320*	*P* < 0.001
		*ER*_*Am*_ = 0.173	*LL* = 0.059	*UL* = *0.413*	*P* < 0.001
		ER_sum_ = 0.222	LL = 0.160	UL = 0.300	*P* < 0.001

*ER* Event rate, *LL* Lower limit, *UL* Upper limit, *Sum* Summary, *Asi* Asia, *Eur* Europe, *Ame* America, *Afr* Africa

**Table 3:** Heterogeneity subgroup meta-analysis of anxiety, distress, depression, sleep problem, insomnia, post-traumatic stress disease (PTSD) based on continents in the 11 studies

**Anxiety**	Continent	*Q*_Ame_ = 398.3189	*df* = 12	*P* < 0.001	*I*^2^ = 96.99
		Q _Afr_ = 0	df = 0	*P* = *1*	*I*^2^ = 0
		*Q*_Asi_ = 9.8038*10^4^	*df* = 270	*P* < 0.001	*I*^2^ = 99.73
		*Q*_Eur_ = 1.5539*10^3^	*df* = 20	*P* < 0.001	*I*^2^ = 98.71
		*Q*_within_ = 9.9990*10^4^	df = 302	*P* < 0.001	*I*^2^ = 99.70
		*Q*_between_ = 1.594	*df* = 3	P = 0.661	
		*Q*_overall_ = 1.0146*10^5^	*df* = 305	*P* < 0.001	*I*^2^ = 99.70
**Distress**	Continent	Q _Asi_ = 3.2398*10^3^	df = 14	*P* < 0.001	*I*^2^ = 99.57
		Q _Eur_ = 132.244	df = 5	*P* < 0.001	*I*^2^ = 96.22
		Q _Ame_ = 0	df = 0	*P* = *1*	*I*^*2*^ = *0*
		Q _Within_ = 3.3721*10^3^	df = 19	*P* < 0.001	I^2^ = 99.44
		Q _between_ = 5.124	df = 2	P = 0.077	
		Q _overall_ = 3.4582*10^3^	df = 21	*P* < 0.001	*I*^2^ = 99.39
**Depression**	Continent	*Q*_Ame_ = 237.97	*df* = 10	*P*<0.001	*I*^2^ = 95.80
		*Q *_Eur_ = 1.130*10^3^	*df* = 19	*P*<0.001	*I*^2^ = 99.32
		Q _Asi_ = 9.7946*10^4^	df = 226	*P*<0.001	*I*^2^ = 99.77
		Q _Afr_ = 0	df = 0	P = 1	*I*^2^ = 0
		*Q*_within_ = 9.9314 *10 ^4^	*df* = 255	*P*<0.001	*I*^2^ = 99.74
		*Q*_between_ = 2.884	*df* = 2	*P* = 0.410	
		*Q*_overall_ = .0244*10^5^	*df* = 3	*P* = 0.012	*I*^2^ = 72.418
**Sleep Problem**	Continent	*Q*_*A*si_ = 9.8119*10^4^	*df* = 132	*P* < 0.001	*I*^2^ = 99.87
		*Q*_*E*ur_ = 4.9220*10^4^	*df* = 43	*P* < 0.001	*I*^2^ = 99.91
		*Q *_*Afr*_ = *523.5537*	df = 7	*P* < 0.001	I^2^ = 98.66
		*Q*_eAme_ = 1.4416*10^3^	*df* = 15	*P* < 0.001	*I*^2^ = 98.96
		*Q*_within_ = 1.4930*10^5^	*df* = 197	*P* < 0.001	*I*^2^ = 99.87
		*Q*_between_ = 37.629	*df* = 3	*P* < 0.001	
		*Q*_overall_ = 1.7992*10^5^	*df* = 200	*P* < 0.001	*I*^2^ = 99.87
**Insomnia**	Continent	*Q*_Asi_ = 2.2492*10^3^	*df* = 20	*P* < 0.001	*I*^2^ = 99.11
		*Q*_Eur_ = 3.1914*10^3^	*df* = 3	*P* < 0.001	*I*^2^ = 99.91
		*Q*_within_ = 1.936	*df* = 23	*P* < 0.001	*I*^2^ = 99.58
		*Q*_between_ = 1.936	*df* = 1	*P* = 0.164	
		*Q*_overall_ = 6.7152*10^3^	*df* = 24	*P* < 0.001	*I*^2^ = 99.64
**PTSD**	continent	*Q *_*Asi*_ = 2.9749*10^*3*^	df = 18	*P* < 0.001	*I*^2^ = 99.40
		*Q *_*Eur*_ = *903/159*	df = 11	*P* < 0.001	I^2^ = 98.78
		*Q *_*Ame*_ = *5.336*	df = 3	*P* = *0.149*	
		*Q*_within_ = 3.8834*10^*3*^	df = 32	*P* < 0.001	I^2^ = 99.18
		*Q*_between_ = *0.993*	df = 2	*P* = *0.609*	
		*Q*_overall_ = 4.0864*10^*3*^	df = 34	*P* < 0.001	*I*^*2*^ = *99.17*

*Asi* Asia, *Eur* Europe, *Ame* America, *Afr* Africa, *df* degree of freedom

### Anxiety

**Fig. 2:**
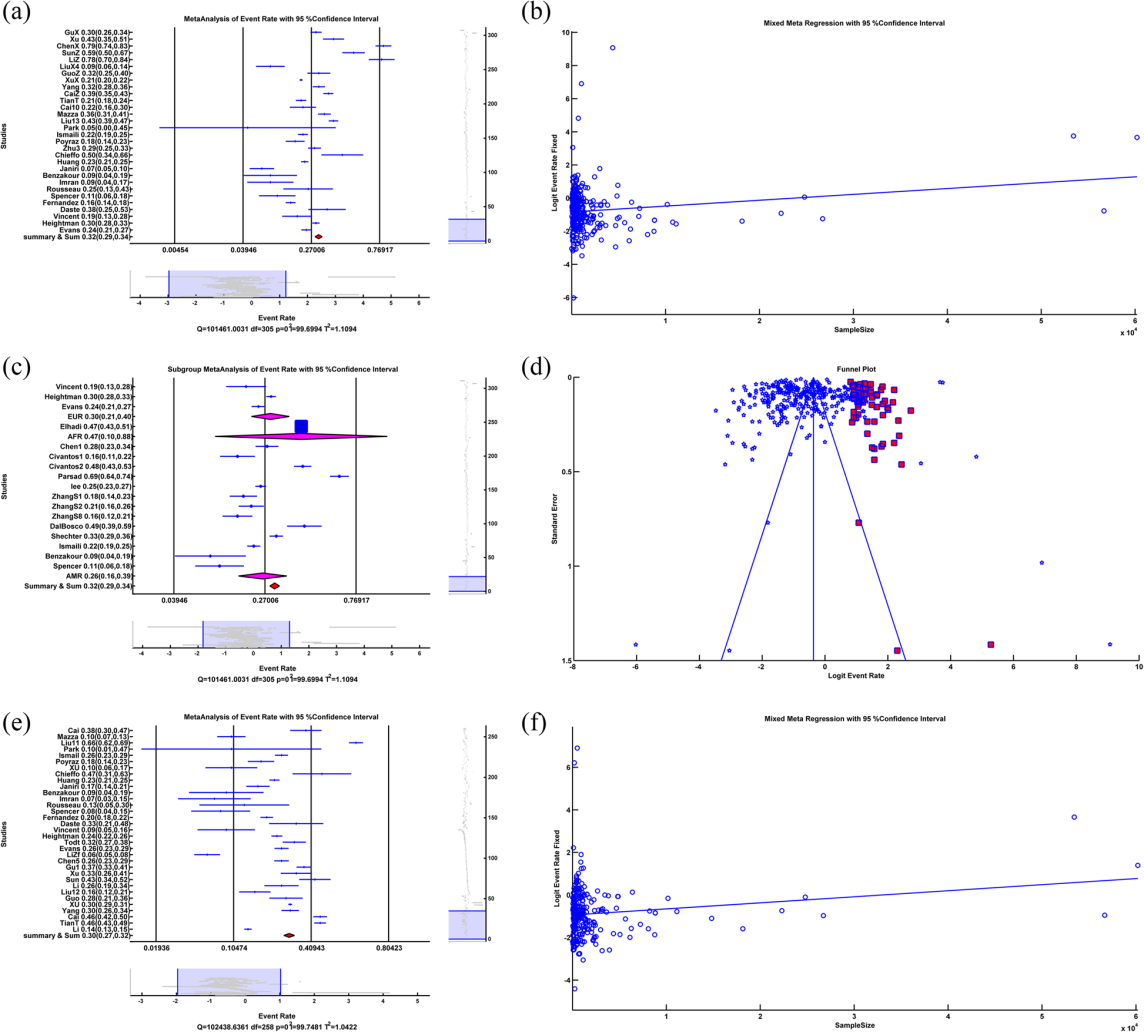
**a** Random based Forest plot of anxiety, **b** Random-effects meta-regression between anxiety rate and sample size, **c** Forest plot of subgroup random-effects meta-analysis of anxiety, **d** Random funnel plot and Trim&Fill in anxiety, **e** Random forest plot of depression, **f** Random-effects meta-regression between depression rate and sample size. (All of the Forest plots are the last representative of the whole forest plot due to the high number of included studies)

The random-effects anxiety meta-analysis showed that ER = 0.318, LL = 0.293, UL = 0.345, *p-value* < 0.001, which means that the anxiety rate due to COVID-19 is 31.8% with a significant *p-value*. The forest plot is illustrated in Fig. 2a. The heterogeneity test showed that Q = 1.0146*10^5^, df = 305, *p-value* < 0.001, I^2^ = 99.70, which denotes a high heterogeneity (99.70%) among studies with significant *p-value*.

We applied a random-effects meta-regression to find the relation between anxiety rate and sample size, as illustrated in Fig. 2b. The results were Slope = 3.53*10^–5^, *p-value* < *0.001.* The positive sign of slope and the significance value *p-value* showed a direct relation between anxiety rate and sample size. And R^2^ = 0%, which meant meta-regression of a sample size could not explain heterogeneity among the studies.

Subgroup random-effects meta-analysis of anxiety due to COVID-19 infection showed that in Asia, ER = 0.323, LL = 0.295, UL = 0.351, *p-value* < 0.001, in Europe, ER = 0.300, LL = 0.210, UL = 0.400, *p-value* < 0.001, in America ER = 0.259, LL = 0.163, UL = 0.385, *p-value* < 0.001, in Africa ER = 0.47, LL = 0.100, UL = 0.876, *p-value* = 0.910 and in the Summary of Continents ER = 0.318, LL = 0.293, UL = 0.345, *p-value* < 0.001 which means the anxiety rate because of Covid-19 in Asia, Europe, America and summary of continents are 32.3%, 30%, 25.9%, and 31.8%, respectively with significant *p-value*. The anxiety rate in Africa was 47%, with an insignificant *p-value.* Insignificant *p-value* in Africa indicated that the difference between the number of people with and without anxiety was negligible. The related forest plot is illustrated in Fig. 2c. The subgroup heterogeneity test showed that in Asia Q = 9.8038*10^4^, df = 270, *p-value* < 0.001, I^2^ = 99.73, in Europe Q = 1.5539*10^3^, df = 20, *p-value* < 0.001, I^2^ = 98.71, in Africa Q = 0, df = 0, *p-value* = *1*, I^2^ = 0, in America Q = 398.3189, df = 12, *p-value* < 0.001, I^2^ = 96.99, Q_within_ = 9.9990*10^4^, df = 302, *p-value* < 0.001, I^2^ = 99.70 and Q_between_ = 1.594, df = 3, *p-value* = 0.661 This meant there was significant heterogeneity in Asia, Europe, America and within continents with 99.73%, 98.71%, 96.99%, and 99.70%, respectively. There was no significant heterogeneity in Africa. Q_between_ and insignificant *p-value* showed that subgroup meta-analysis couldn't explain the source of heterogeneity among the studies.

Eggers' test as a publication bias test showed that Intercept = -1.343, *p-value* = 0.390. The insignificant *p-value* didn't prove publication bias among the studies, but the funnel plot showed asymmetry. Therefore, we applied a Trim&Fill operation illustrated in Fig. 2d. After applying random Trim&Fill procedure, 69 new studies were found. By adding 69 additional studies to the studies and using a random-effects meta-analysis, the results were as ER = 0.409, LL = 0.381, UL = 0.438, *p-value* < 0.001. By adding 69 new studies, 40.9% of the people had anxiety problems due to COVID-19 with significant *p-value.* The heterogeneity test showed that Q = 1.3565*10^5^, df = 374, *p-value* < 0.001, I^2^ = 99.72, which means a high heterogeneity (99.72%) among studies with significant *p-value*.

### Depression

Random-effects meta-analysis of depression showed that ER = 0.295, LL = 0.270, UL = 0.322, *p-value* < 0.001, which means that the depression rate due to Covid-19 is 29.5% with a significant *p-value*. The forest plot is illustrated in Fig. 2e. The heterogeneity test showed that Q = 1.0244*10^5^, df = 258, *p-value* < 0.001, I^2^ = 99.75, which means a high heterogeneity (99.75%) among studies with significant *p-value*.

We applied a random-effects meta-regression to find the relation between depression rate and sample size, illustrated in Fig. 2f. The results were slope = 2.851*10^–5^, *p-value* = *0.001.* The positive sign of slope and the significant *p-value* showed a direct relationship between depression rate and sample size. And R^2^ = 12.827%, which meant that meta-regression of a sample size could explain 12.827% of heterogeneity among the studies.

**Fig. 3:**
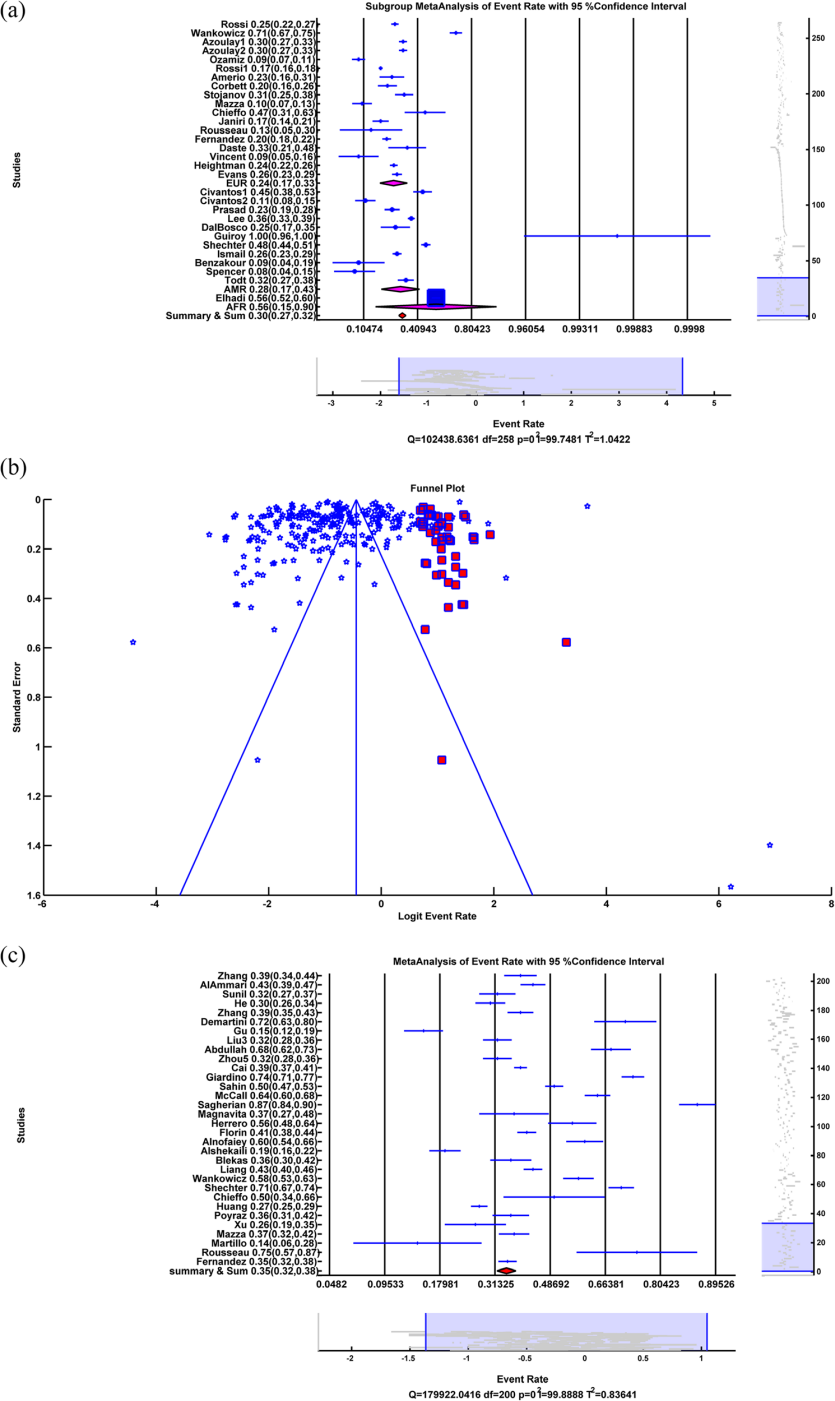
**a** Forest plot of Subgroup random-effects meta-analysis of depression, **b** Funnel plot and Trim&Fill in anxiety, **c** Forest plot of Sleep Problem. (All of the Forest plots are the last representative of the whole forest plot due to the high number of included studies)

Subgroup Random meta-analysis of depression due to COVID-19 showed that in Asia ER = 0.300, LL = 0.273, UL = 0.329, *p-value* < 0.001, in Europe ER = 0.239, LL = 0.166, UL = 0.331, *p-value* < 0.001, in America ER = 0.283, LL = 0.173, UL = 0.426, *p-value* = 0.004, in Africa ER = 0.56, LL = 0.148, UL = 0.904, *p-value* = 0.813 and in Summary of Continents ER = 0.295, LL = 0.270, UL = 0.322, *p-value* < 0.001 which meant the depression rate due to COVID-19 in Asia, Europe, America and the summary of continents was 30%, 23.9%, 28.3%, and 29.5% respectively with significant *p-value*. The depression rate in Africa was 56%, with an insignificant *p-value.* The insignificant *p-value* in Africa indicated that the difference between the number of people with depression and those without depression was insignificant. The related forest plot is illustrated in Fig. 3a. The subgroup heterogeneity test showed that in Asia Q = 9.7946*10^4^, df = 226, *p-value* < 0.001, I^2^ = 99.77, in Europe Q = 1.1302*10^3^, df = 19, *p-value* < 0.001, I^2^ = 99.32, in Africa Q = 0, df = 0, *p-value* = *1*, I^2^ = 0, in America Q = 237.97, df = 10, *p-value* < 0.001,I^2^ = 95.80 Q_within_ = 9.9314*10^4^, df = 255, *p-value* < 0.001, I^2^ = 99.74 and Q_between_ = 2.884, df = 3, *p-value* = 0.410. It meant that there was significant heterogeneity in Asia, Europe, the US, and within continents, with 99.77%, 99.32%, 95.80%, and 99.74%, respectively. There was no significant heterogeneity in Africa. Q_between_ and insignificant *p-value* showed that subgroup meta-analysis couldn't explain the source of heterogeneity among the studies.

Eggers' test as a publication bias test showed that Intercept = -5.723, *p-value* = 0.001. A significant p-value proves publication bias among the studies, and the funnel plot showed asymmetry, so we applied a random Trim&Fill method illustrated in Fig. 3b. After applying random Trim&Fill, 49 new studies were found. By adding 49 additional studies to the studies and using a random-effects meta-analysis, the results were ER = 0.364, LL = 0.337, UL = 0.392, *p-value* < 0.001. By adding 49 new studies, 36.4% of people have depression problems due to COVID-19 with significant *p-value.* The heterogeneity test showed that Q = 1.1521 *10^5^, df = 307, *p-value* < 0.001, I^2^ = 99.73, which meant a high heterogeneity (99.73%) among studies with significant *p-value*.

### Sleep problem

The random-effects meta-analysis of sleep problems showed that ER = 0.347, LL = 0.318, UL = 0.376, *p-value* < 0.001, which means that sleep problems resulting from COVID-19 are 34.6% with a significant *p-value*. The forest plot is illustrated in Fig. 3c. The heterogeneity test showed that Q = 1.7992*10^5^, df = 200, *p-value* < 0.001, I^2^ = 99.89, which may indicate a high heterogeneity (99.89%) among studies with significant *p-value*.

**Fig. 4:**
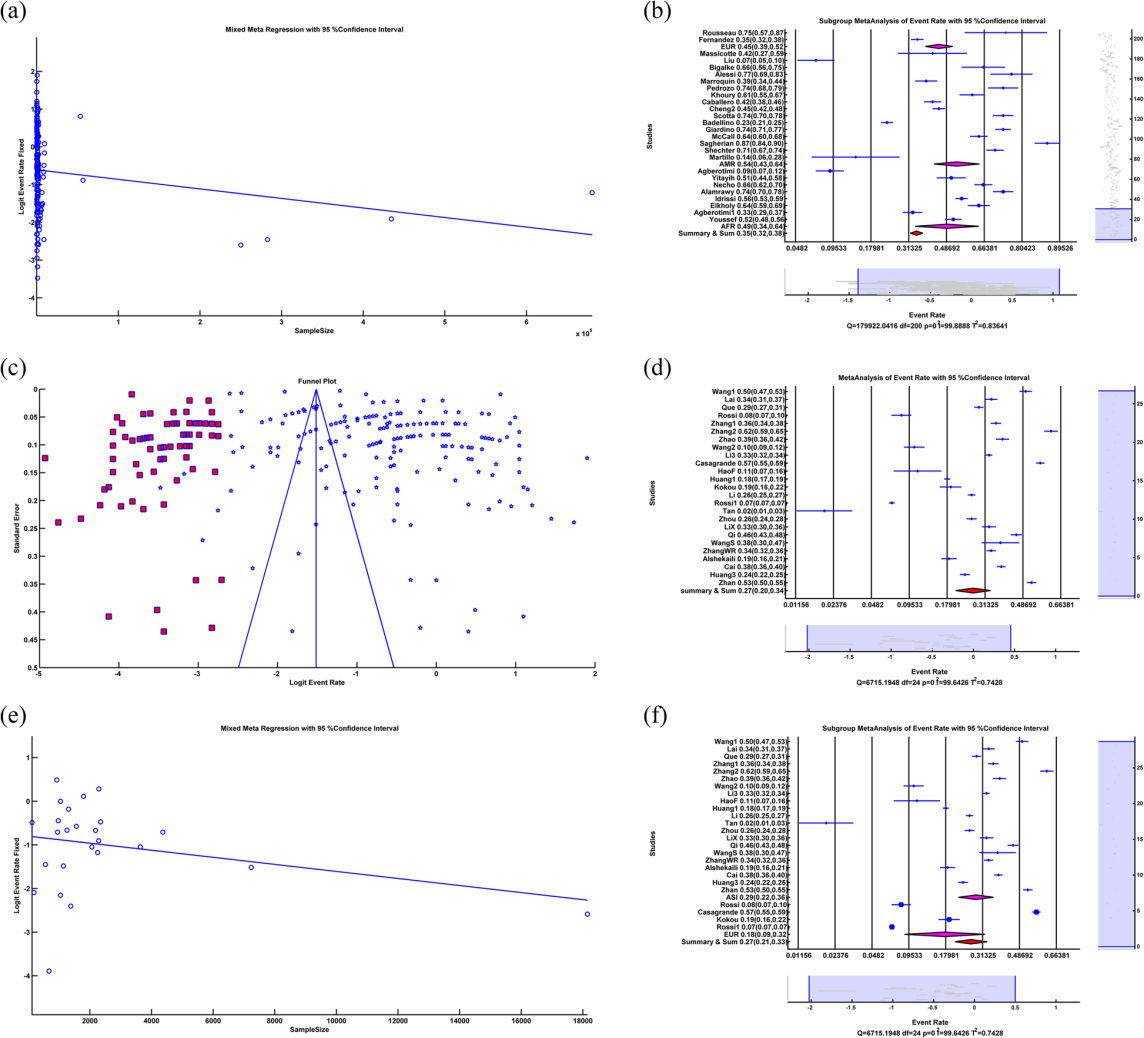
**a** Random-effects meta-regression between Sleep Problem rate and sample size, **b** Forest plot of Subgroup random-effects meta-analysis of Sleep Problem, **c** Funnel plot and Trim&Fill in Sleep Problem, **d** Forest plot of insomnia, **e** Random-effects meta-regression between Insomnia rate and sample size, **f** Forest plot of Subgroup random-effects meta-analysis of insomnia. (All of the Forest plots are the last representative of the whole forest plot due to the high number of included studies)

We applied a random-effects meta-regression to find the relation between sleep problem rate and sample size illustrated in Fig. 4a. The results were slope = -2.529*10^–6^, *p-value* = *0.029.* The negative sign of slope and the significant *p-value* showed an inverse relationship between sleep problem rate and sample size. And R^2^ = 0% meant meta-regression of a sample size could not explain heterogeneity among the studies.

The subgroups of random-effects meta-analysis of sleep problems due to COVID-19 showed that in Asia ER = 0.288, LL = 0.258, UL = 0.321, *p-value* < 0.001, in Europe ER = 0.451, LL = 0.386, UL = 0.517, *p-value* = 0.145, in America ER = 0.536, LL = 0.426, UL = 0.643, *p-value* = 0.523, in Africa ER = 0.489, LL = 0.34, UL = 0.64, *p-value* = 0.89 and in the Summary of Continents ER = 0.347, LL = 0.319, UL = 0.375, *p-value* < 0.001 which meant the rate of sleep problem due to Covid-19 in Asia, and summary of continents were 28.8% and 34.7% respectively with significant *p-value*. The sleep problem rate in Europe, Africa and America were 45.1%, 48.9%, and 53.6%, respectively, with an insignificant *p-value.* The insignificant *p-value* in Europe, Africa, and America indicated that the number of people with sleep problems and those without sleep problems was not significant. The related forest plot is illustrated in Fig. 4b. The subgroup heterogeneity test showed that in Asia Q = 9.8119*10^4^, df = 132, *p-value* < 0.001, I^2^ = 99.87, in Europe Q = 4.9220*10^4^, df = 43, *p-value* < 0.001, I^2^ = 99.91, in Africa Q = 523.5537, df = 7, *p-value* < *0.001*, I^2^ = 98.66, in America Q = 1.4416*10^3^, df = 15, *p-value* < 0.001, I^2^ = 98.96 Q_within_ = 1.4930*10^5^, df = 197, *p-value* < 0.001, I^2^ = 99.87 and Q_between_ = 37.629, df = 3, *p-value* < *0.001* This meant there was significant heterogeneity in Asia, Europe, America, Africa and within continents with 99.87%, 99.91%, 98.96%, 98.66% and 99.87% respectively. Q_between_ and significant *p-value* showed that subgroup meta-analysis could explain R^2^ = 5.691% of the source of heterogeneity among the studies.

Egger’s test as a publication bias test showed Intercept = 10.106, *p-value* < 0.001. A significant *p-value* proved publication bias among studies, and the funnel plot showed asymmetry, so we applied a random Trim&Fill method illustrated in Fig. 4c. After using the random Trim&Fill operation, 27 new studies were found. By adding these newly found studies to the studies and applying a random effect meta-analysis, the results were ER = 0.292, LL = 0.267, UL = 0.318, *p-value* < 0.001. By adding 27 more studies, 29.2% of people have sleep problems due to COVID-19 with significant *p-value.* The heterogeneity test showed that Q = 2.0245*10^5^, df = 227, *p-value* < 0.001, I^2^ = 99.89, which meant a high heterogeneity (99.89%) among studies with significant *p-value*.

### Insomnia

The random-effects meta-analysis of insomnia showed that ER = 0.265, LL = 0.205, UL = 0.337, *p-value* < 0.001 insomnia due to Covid-19 is 26.5% with a significant *p-value*. The forest plot is illustrated in Fig. 4d. The heterogeneity test showed that Q = 6.7152*10^3^, Df = 24, *p-value* < 0.001, I^2^ = 99.64, which may reveal a high heterogeneity (99.64%) among studies with significant *p-value*.

We applied a random-based meta-regression to find the relation between the insomnia rate and the sample size illustrated in Fig. 4e. The results were slope = -8.0835*10^–5^, *p-value* = *0.011.* The negative sign of slope and the significant *p-value* showed an inverse relation between insomnia rate and sample size. And R^2^ = 58.224%, which means that meta-regression of a sample size could explain 58.224% of heterogeneity among the studies.

Subgroup random-effects meta-analysis of insomnia due to Covid-19 showed that in Asia ER = 0.286, LL = 0.22, UL = 0.362, *p-value* < 0.001, in Europe ER = 0.177, LL = 0.088, UL = 0.324, *p-value* < 0.001 and in the summary of Continents ER = 0.266, LL = 0.208, UL = 0.333, *p-value* < 0.001 which meant that the insomnia rate because of Covid-19 in Asia, Europe and summary of continents were 28.6%, 17.7% and 26.6% respectively with significant *p-value*. The related forest plot is illustrated in Fig. 4f. The subgroup heterogeneity test showed that in Asia Q = 2.2492*10^3^, df = 20, *p-value* < 0.001, I^2^ = 99.11, in Europe Q = 3.1914*10^3^, df = 3, *p-value* < 0.001, I^2^ = 99.91, Q_within_ = 1.936, df = 23, *p-value* < 0.001, I^2^ = 99.58 and Q_between_ = 1.936, df = 1, *p-value* = 0.164. It meant that there was significant heterogeneity in Asia, Europe, and within continents, with 99.11%, 99.91%, 99.58% and 99.64%, respectively. Q_between_ and insignificant *p-value* showed that subgroup meta-analysis couldn’t explain the source of heterogeneity among the studies.

**Fig. 5:**
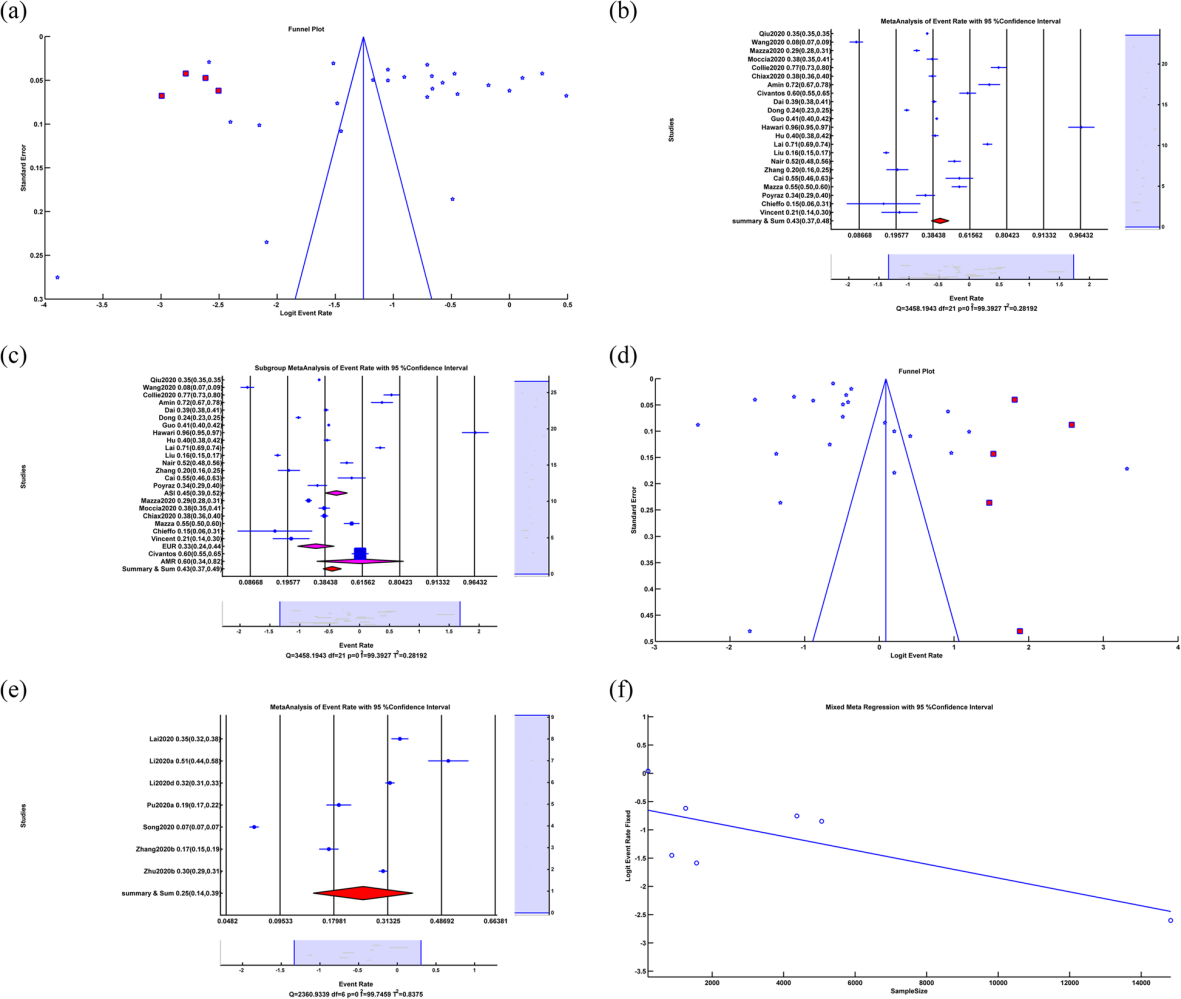
**a** Funnel plot and Trim&Fill in Insomnia, **b** Forest plot of distress, **c** Forest plot of subgroup random-effects meta-analysis of distress, **d** Funnel plot and Trim&Fill in Distress, **e** Forest plot of PTSS, **f** Random-effects meta-regression between PTSS rate and sample size. (All of the Forest plots are the last representative of the whole forest plot due to the high number of included studies)

The Egger’s test as a publication bias test showed that Intercept = 5.527, *p-value* = 0.505. Insignificant *p-value* could not prove publication bias among the studies, and the funnel plot showed asymmetry among studies, so we applied a random Trim&Fill method illustrated in Fig. 5a. After applying random Trim&Fill, four new studies were found. By adding four additional studies to the studies and using a random-effects meta-analysis, the results were ER = 0.221, LL = 0.164, UL = 0.291, *p-value* < 0.001. By adding four new studies, 22.1% of people have insomnia problems because of Covid-19 with significant *p-value.* The heterogeneity test showed that Q = 1.0698 *10^4^, df = 28, *p-value* < 0.001, I^2^ = 99.74, which means there was a high (99.74%) heterogeneity among studies with significant *p-value*.

### Distress

The random-effects meta-analysis of distress showed that ER = 0.428, LL = 0.373, UL = 0.485, *p-value* = 0.013, which means that distress due to Covid-19 is 42.8% with a significant *p-value*. A significant *p-value* indicated that the difference between the number of people with distress problems and those without distress problems was insignificant. The forest plot is illustrated in Fig. 5b. The heterogeneity test showed that Q = 3.4582*10^3^, df = 21, *p-value* < 0.001, I^2^ = 99.39, which meant that there was a high heterogeneity (99.39%) among studies with significant *p-value*.

We applied a random-based meta-regression to find the relation between distress rate and sample size. The results were Slope = -1.115*10^–5^, *p-value* = *0.422.* The insignificant *p-value* did not show a relationship between distress rate and sample size. The meta-regression of a sample size could not explain the source of heterogeneity among studies.

Subgroup random-effects meta-analysis of distress because of COVID-19 showed that in Asia, ER = 0.454, LL = 0.385, UL = 0.524, *p-value* = 0.199; in Europe, ER = 0.332, LL = 0.238, UL = 0.441, *p-value* = 0.003, in America ER = 0.602, LL = 0.336, UL = 0.819, *p-value* = 0.460 and in the Summary of Continents ER = 0.428, LL = 0.372, UL = 0.487, *p-value* = 0.016 which meant that the distress rate because of Covid-19 in Asia, Europe, America and summary of continents were 45.4%, 33.2%, 60.2%, and 42.8% respectively. An insignificant p-value indicated that the difference between the number of people with distress and those without distress was not significant. The related forest plot is illustrated in Fig. 5c. The subgroup heterogeneity test showed that in Asia Q = 3.2398*10^3^, df = 14, *p-value* < 0.001, I^2^ = 99.57, in Europe Q = 132.244, df = 5, *p-value* < 0.001, I^2^ = 96.22, in America Q = 0, df = 0, *p-value* = 1, I^2^ = 0, Q_within_ = 3.3721*10^3^, df = 19, p < 0.001, I^2^ = 99.44 and Q_between_ = 5.124, df = 2, *p-value* = 0.077 This meant there was significant heterogeneity in Asia, Europe, and within continents with 99.57%, 96.22% and 99.44% respectively. There was no significant heterogeneity in America. Q_between_ and insignificant *p-value* showed that subgroup meta-analysis couldn't explain the source of heterogeneity among the studies.

Eggers' test as a publication bias test showed that Intercept = 3.463, *p-value* = 0.354. Insignificant *p-value could not* prove publication bias among the studies, and the funnel plot showed asymmetry among studies, so we applied a random Trim&Fill method illustrated in Fig. 5d. After applying random Trim&Fill, five new studies were found. By adding five new studies to the studies and using a random-effects meta-analysis, the results were ER = 0.522, LL = 0.445, UL = 0.597, *p-value* < 0.579. By adding five more studies, 52.2% of people have distress problems due to COVID-19 with insignificant *p-value.* An insignificant *p-value* indicated that the difference between the number of people with distress and those without distress problems was not significant. The heterogeneity test showed that Q = 8.4909*10^3^, df = 26, *p-value* < 0.001, I^2^ = 99.69, which means a high (99.69%) heterogeneity among studies with significant *p-value*.

### Post-traumatic stress syndrome (PTSS)

The random-effects meta-analysis of PTSS showed that ER = 0.246, LL = 0.142, UL = 0.392, *p-value* = 0.001, which means PTSS because of COVID-19 is 24.6% with a significant *p-value*. The forest plot is illustrated in Fig. 5e. The heterogeneity test showed that Q = 2.3609*10^3^, df = 6, *p-value* < 0.001, I^2^ = 99.75, which means a high (99.75%) heterogeneity among studies with significant *p-value*.

We applied a random-based meta-regression to find the relation between the PTSS rate and the sample size illustrated in Fig. 5f. The results were Slope = -1.225*10^–4^, *p-value* = *0.002.* The negative sign of slope and the significant *p-value* showed an inverse relation between the PTSS rate and the sample size. And R^2^ = 71.89%, which meant that meta-regression of a sample size could explain 71.89% of heterogeneity among the studies.

We could not apply a subgroup meta-analysis because all the studies were Asian.

**Fig. 6:**
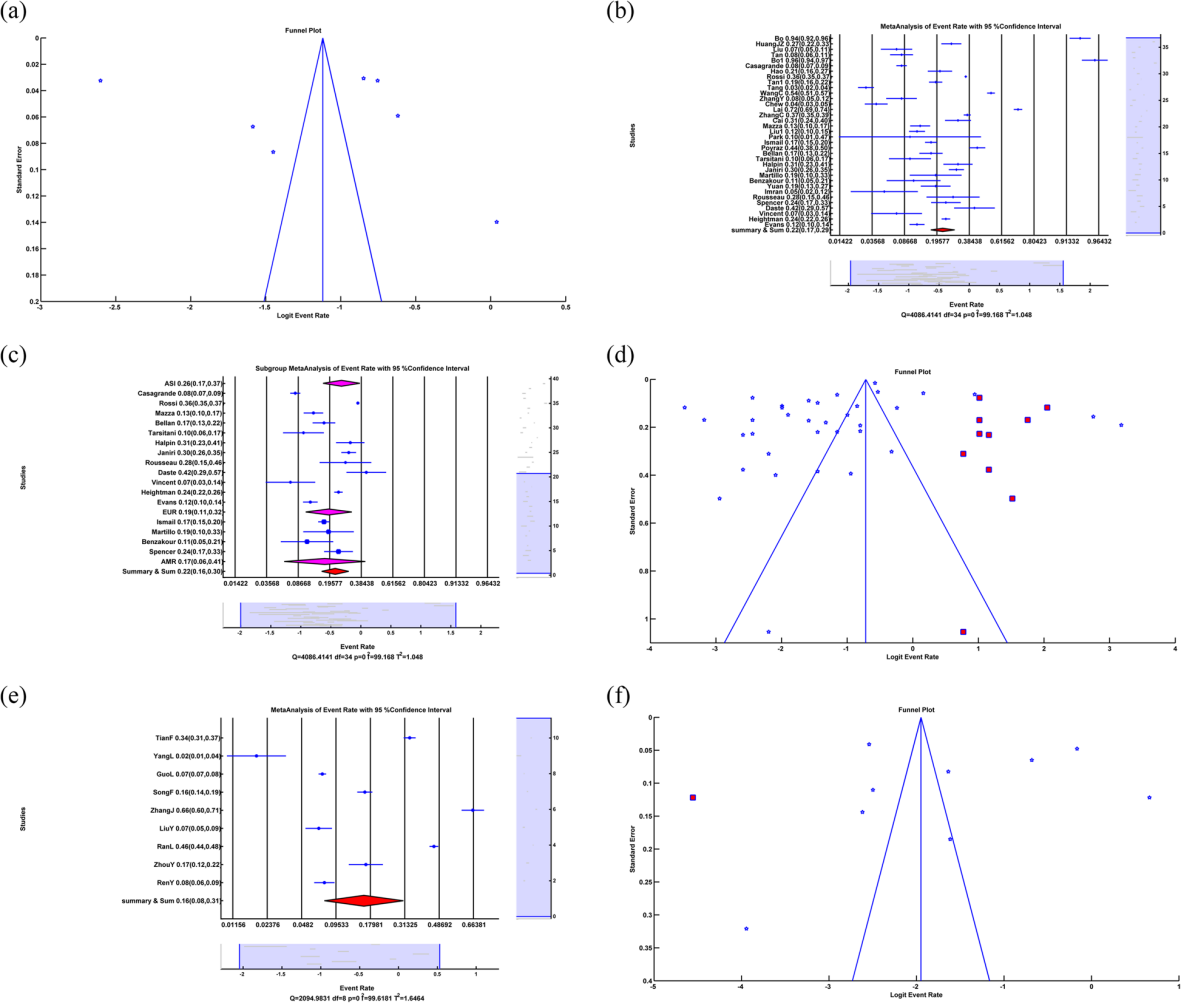
**a** Funnel plot in PTSS, **b** Forest plot of PTSD, **c** Forest plot of Subgroup random-effects meta-analysis of PTSD, **d** Funnel plot and Trim&Fill in PTSD, **e** Forest plot of somatic symptoms, **f** Funnel plot and Trim&Fill in Somatic Symptoms. (All of the Forest plots are the last representative of the whole forest plot due to the high number of included studies)

Eggers' test as a publication bias test showed that Intercept = 7.005, *p-value* = 0.728. An insignificant *p-value* couldn't prove publication bias among the studies, and the funnel plot did not show asymmetry, which is illustrated in Fig. 6a.

### PTSD

Random-effects meta-analysis of PTSD showed that ER = 0.223, LL = 0.169, UL = 0.29, *p-value* < 0.001, which means PTSD because of COVID-19 is 22.3% with a significant *p-value*. The forest plot is illustrated in Fig. 6b. The heterogeneity test showed that Q = 4.0864*10^3^, df = 34, *p-value* < 0.001, I^2^ = 99.17, which means that there was a high heterogeneity (99.17%) among studies with significant *p-value*.

We applied a random-based meta-regression to find the relation between PTSD rate and sample size. The results were slope = 3.624*10^–5^, *p-value* = *0.63.* The insignificant *p-value* did not show an association between the rate of PTSD and sample size. Meta-regression of a sample size couldn't explain the source of heterogeneity among studies.

The subgroups of random-effects meta-analysis of PTSD due to COVID-19 showed that in Asia, ER = 0.257, LL = 0.166, UL = 0.375, *p-value* < 0.001, in Europe, ER = 0.191, LL = 0.106, UL = 0.320, *p-value* < 0.001, in America ER = 0.173, LL = 0.059, UL = 0.413, *p-value* < 0.001 and in the Summary of Continents ER = 0.222, LL = 0.160, UL = 0.300, *p-value* < 0.001 which meant the PTSD rate due to COVID-19 in Asia, Europe, America and summary of continents was 25.7%, 19.1%, 17.3% and 22.2% respectively. A significant *p-value* indicated that the difference between the number of people with PTSD and those without PTSD was significant. The related forest plot is illustrated in Fig. 6c. The subgroup heterogeneity test showed that in Asia Q = 2.9749*10^3^, df = 18, *p-value* < 0.001, I^2^ = 99.40, in Europe Q = 903.159, df = 11, *p-value* < 0.001, I^2^ = 98.78, in America Q = 5.336, df = 3, *p-value* = 0.149, Q_within_ = 3.8834*10^3^, df = 32, *p-value* < 0.001, I^2^ = 99.18 and Q_between_ = 0.993, df = 2, *p-value* = 0.609 This meant that there was significant heterogeneity in Asia, Europe and within continents with 99.40%, 99.78% and 99.18% respectively. There was insignificant heterogeneity in America, with 43.78% with Q_between,_ and the insignificant *p-value* showed that subgroup meta-analysis could not explain the source of heterogeneity among the studies.

Eggers' test as a publication bias test showed that Intercept = -4.239, *p-value* = 0.078. Insignificant *p-value could not* prove publication bias among the studies, and the funnel plot showed asymmetry among studies, so we applied a random Trim&Fill method illustrated in Fig. 6d. After applying random Trim&Fill, ten new studies were found. By adding ten additional studies to the studies and using a random effect meta-analysis, the results were ER = 0.328, LL = 0.259, UL = 0.406, *p-value* < 0.001. This meant that by adding ten new studies, 32.8% of people have PTSD problems due to COVID-19 with significant *p-value.* A significant *p-value* indicated that the difference between the number of people with PTSD problems and those without PTSD problems was significant. The heterogeneity test showed that Q = 5.5245 *10^3^, df = 44, *p-value* < 0.001, I^2^ = 99.20, which means a high (99.20%) heterogeneity among studies with significant *p-value*.

### Somatic symptoms

The random-effects meta-analysis of somatic symptoms showed that ER = 0.16, LL = 0.076, UL = 0.307, *p-value* < 0.001, which means that somatic symptoms due to COVID-19 is 16% with significant *p-value*. The forest plot is illustrated in Fig. 6e. The heterogeneity test showed that Q = 2.095*10^3^, df = 8, *p-value* < 0.001, I^2^ = 99.62, which means that there was a high heterogeneity (99.62%) among studies with significant *p-value*.

We applied a random-based meta-regression to find the relation between the rate of somatic symptoms and sample size. The results were Slope = -1.09*10^–4^, *p-value* = *0.463.* The insignificant *p-value* did not show a connection between the rate of somatic symptoms and the sample size. Meta-regression of a sample size could not explain the source of heterogeneity among studies.

We could not apply a subgroup meta-analysis because all the studies were Asian.

Eggers' test as a publication bias test showed that Intercept = -0.768, *p-value* = 0.949. Insignificant *p-value* could not prove publication bias among the studies, and the funnel plot showed asymmetry among studies, so we applied a random Trim&Fill method illustrated in Fig. 6f. After applying random Trim&Fill, one new study was found. By adding one study to the studies and using a random effect meta-analysis, the results were ER = 0.125, LL = 0.056, UL = 0.257, *p-value* < 0.001. This meant that by adding one new study, 12.5% of people somatic symptom problems because of COVID-19 with significant *p-value.* The heterogeneity test showed that Q = 2.729 *10^3^, df = 9, *p-value* < 0.001, I^2^ = 99.67, which means there was a high (99.67%) heterogeneity among studies with significant *p-value*.

### Fear symptom

**Fig. 7:**
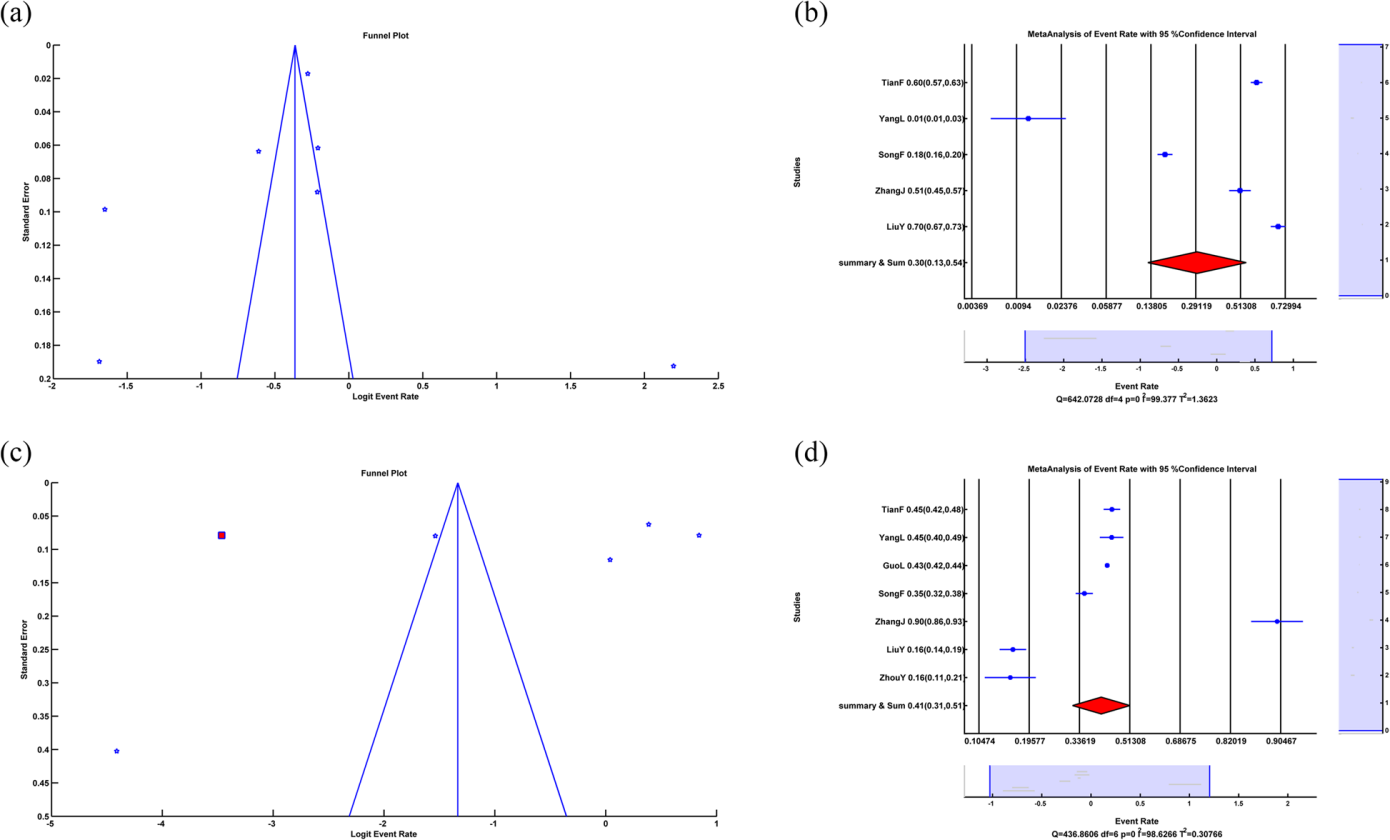
**a** Forest plot of Fear Symptom, **b** Funnel plot in Fear Symptom, **c** Forest plot of Obsessive Compulsive symptom, **d** Funnel plot and Trim&Fill in Obsessive Compulsive symptom. (All of the Forest plots are the last representative of the whole forest plot due to the high number of included studies)

A random-effects meta-analysis of fear symptoms showed that ER = 0.41, LL = 0.313, UL = 0.514, *p-value* = 0.089, which means fear symptoms because of COVID-19 is 41% with insignificant *p-value*. An insignificant *p-value* indicated that the difference between the number of people with fear symptoms and those without fear symptoms was not significant. The forest plot is illustrated in Fig. 7a. The heterogeneity test showed that Q = 436.861, df = 6, *p-value* < 0.001, I^2^ = 98.63, which meant that there was a high heterogeneity (98.63%) between studies with significant *p-value*.

We applied a random-based meta-regression to find the relation between fear-symptom rate and sample size. The results were Slope = 3.657*10^–6^, *p-value* = *0.957.* The insignificant *p-value* did not show a link between fear symptom rate and sample size. So, meta-regression of a sample size could not explain the source of heterogeneity among studies.

We could not apply a subgroup meta-analysis because all the studies were Asian.

Egger’s test as a publication bias test showed that Intercept = -2.016, *p-value* = 0.701. An insignificant *p-value* could not prove publication bias among the studies, and the funnel plot showed no asymmetry, which is illustrated in Fig. 7b.

### Obsessive–compulsive symptoms

The random-effects meta-analysis of obsessive–compulsive symptoms showed that ER = 0.297, LL = 0.130, UL = 0.543, *p-value* = *0.103,* which means obsessive–compulsive symptoms due to COVID-19 is 29.67% with insignificant *p-value*. The forest plot is illustrated in Fig. 7c. The insignificant *p-value* indicated that the difference between the number of people with fear and those without fear symptoms was not significant. The heterogeneity test showed that Q = 642.073, df = 4, *p-value* < 0.001, I^2^ = 99.38, which means that there was a high heterogeneity (99.38%) among studies with significant *p-value*.

We applied a random meta-regression to find the relationship between obsessive–compulsive symptom rate and sample size. The results were slope = 0.001, *p-value* = *0.551.* The insignificant *p-value* did not show an association between the rate of obsessive–compulsive symptom rate and the sample size. And, meta-regression of a sample size could not explain the source of heterogeneity among the studies.

We could not apply a subgroup meta-analysis because all the studies were Asian.

Eggers' test as a publication bias test showed that Intercept = -12.893, *p-value* = 0.437. Insignificant *p-value* could not prove publication bias among the studies, and the funnel plot showed asymmetry among studies, so we applied a random Trim&Fill method illustrated in Fig. 7d. After applying random Trim&Fill, one new study was found. By adding one new study to the studies and using a random effect meta-analysis, the results were ER = 0.208, LL = 0.057, UL = 0.536, *p-value* = 0.077. By adding a study, 20.8% of people have obsessive–compulsive symptoms due to COVID-19 with insignificant *p-value.* The insignificant *p-value* indicated that the difference between the number of people with obsessive–compulsive symptoms and those without obsessive–compulsive symptoms was not significant. The heterogeneity test showed that Q = 2.145 *10^3^, df = 5, *p-value* < 0.001, I^2^ = 99.77 which meant there was a high heterogeneity (99.77%) among studies with significant *p-value*.

## Discussion

Eleven meta-analysis studies evaluated the clinical psychological consequences of COVID-19 disease published between 2019 and 2022. Current meta-analysis reports event rates of anxiety, depression, distress (ER = 0.318 ER = 0.295 ER = 0.428), sleep problems (ER = 0.347), insomnia (ER = 0.265) PTSS (ER = 0.246), PTSD (ER = 0.223), obsessive–compulsive Sympltom(0.297), fear (0.41) and somatic symptoms (ER = 0.16). The highest prevalence of psychiatric disorders is fear and the highest prevalence of psychological symptoms is distress, while the lowest prevalence of psychiatric disorders is PTSD (ER = 0.223) and psychological symptoms is Somatic symptoms (ER = 0.16). Among the results of meta-analysis studies of the literature, there were also high levels of heterogeneity and variation in the conditions mentioned for patients with COVID-19 in two recent years around the world. The results of 11 selected meta-analysis studies were the same across studies. Risks of bias and high heterogeneity within included meta-analysis had the same conclusions drawn from data synthesis.

The I^2^ values describe the percentage of total variation and heterogeneities in the included meta-analysis studies. This current meta-analysis has high statistical heterogeneity in the different symptoms and disorders of the COVID-19 study.

As a result of COVID-19's psychological effects, patients suffer from psychiatric disorders (OCS, PTSD, fear symptoms and phobia, sleep disorders, and PTSS), and psychological symptoms such as insomnia, somatic symptoms, depression, anxiety, distress.

There may be some understanding of COVID-19's behavioral response [64]. Various mental health conditions have also shown that fear of death plays a causal role in the prediction of psychologic symptoms, including COVID-19 [65–68]. By treating death psychologic symptoms directly, current standard treatments may be able to prevent deaths often encountered in mental health services [69].

Some authors studied the psychopathological effect of COVID-19 infection as follows.

Dong et al. report the event rates of the prevalence of different psychological conditions (anxiety, sleep problems, somatic, fear, and obsessive–compulsive symptoms) during the pandemic of COVID-19 [25]. Ping Sun [27] studied the prevalence of anxiety, depression, and insomnia with subgroup analysis ability. Kavita Batra [33] examined the prevalence of anxiety, depression, insomnia, distress, sleep impairment, and post-traumatic stress disorders (PTSD by assessing publication bias and Egger's test indices.

Rajesh Kumar et al. [70] studied the prevalence of psychological outcomes such as stress, depression, anxiety, sleep disturbances, distress, suicidal ideas, use of illegal drugs, and impaired mental health with the determination of heterogeneity. Cenat et al. [20] studied the prevalence of depression, anxiety, insomnia, post-traumatic stress disorder, psychological distress, and mental health disorders.

Silva et al. studied the prevalence of anxiety, depression, and insomnia with the determination of publication bias [31]. Alimoradi et al. [29] investigated sleep problems, depression, and anxiety with subgroup analysis, meta-regression, and publication bias determination. Ching et al. [28] studied the prevalence of psychological distress, such as depression, anxiety, stress, fear, and burnout, and determined heterogeneity and publication bias.

In the literature, Geovan Menezes conducted only one meta-review of Meta-analysis study [60] was performed by Geovan Menezes with the inclusion of 18 meta-analysis with the results of 31.99% and I^2^ = 99.9% for psychological stress, 37.74% with I^2^ = 99.7% for the population of health care workers.

In the study of Menezes, the prevalence and ER of insomnia, distress, and stress was 32.4%, 36%, and 31.99%, and anxiety, depression and PTSD were 27.77%, 26.93%, and 20%. While event rates of anxiety, depression, stress, sleep problems, insomnia, distress, PTSS, PTSD, somatic, fear and obsessive–compulsive symptoms were: 31.8%. 29.5%, 30.4%, 34.6%, 26.5%,42.8%, 24.6%, 22.3%, 16%, 41%, 29.7%.

The random-effects meta-analysis of all outcomes of the current study has had a high heterogeneity, which could not be explained by meta-regression in anxiety, stress, sleep apnea, distress, PTSD, somatic, fear, and obsessive–compulsive symptoms. Still, it could be defined by other outcomes such as insomnia, depression, and PTSS (71.89%). A subgroup meta-analysis was performed between the continents of Asia, Europe, America and Africa, and the heterogeneities were explained in anxiety and sleep problems (5.69%). Other outcomes, such as insomnia, distress, PTSD, and depression, could not be explained. Egger tests to detect publishing bias could not prove any publication bias in the results of anxiety, stress, insomnia, distress, PTSS, PTSD, somatic, fear and obsessive–compulsive symptoms, but could prove depression and sleep problems. Trim and fill operations were performed when there was an asymmetry in the funnel plots. High heterogeneities were present in all 10 outcomes.

Geovan Menezes’ study has not performed trim and fill operations and compared the (Health care worker) HCW people with higher heterogeneity in stress than the general population. This comparison may not be possible in the current study due to a lack of comparison data.

The funnel plots of Menezes's study have also shown asymmetry. The prevalence and ER of depression, anxiety, and stress in Menezes’ study are 26.94%, 27.77%, and 31.94%.

In the current meta-analysis, the included results of the studies may have excessive clinical diversity, so it is not feasible to estimate the overall effect based on the high degree of heterogeneity. Although high in I^2^, Meta-analysis still has low predictive values in high heterogeneity, which is why most meta-analyses have low predictive values. And in situations of high heterogeneities and I^2^ ≥ 50, the random effect model should be used and performed in all steps of the current study [71].

The strength points of the current study are AMSTAR 2 that evaluates and qualifies the included studies in the results and discusses their interpretations.

In addition to aggregating and analyzing the results of individual studies, meta-analysis can factor in heterogeneity, publication bias, meta-regression, and subgroup analysis within continents. Clinical implications of psychiatric symptoms of COVID 19 are isolation from work loss and loneliness, financial stability, grief, multi-organ failure (liver lung kidney and brain) with symptoms lasting weeks months, even years after COVID-19 infection, permanent fear obsession, and suicide as described in the Introduction section completely [72].

The limitations of the current study are the Menezes's, which showed higher heterogeneity. Lack of RCT studies in the present study. The main limitation is the biases that affect primary studies, duplication, and reporting and selection biases. There are several factors that can contribute to the invalidity of a meta-analysis, including the failure to account for important covariants and an overestimation of the results' strength and precision. The pros and cons of meta-analysis are (i) Heterogeneity between studies and their results should be explaine,. (ii) it may reduce the probability of false adverse effects, (iii) allows for an objective appraisal of evidence, (iv) it can estimate the pooled effect, and (v) above-mentioned clinical implications may also be important factors.

Any variability among studies in a systematic review may have heterogeneity, called clinical diversity [55]. High heterogeneity can also occur because two or more symptoms in the studies have different actual effects [55]. Such information can be valuable for researchers because it might allow us to find specific contexts which undoubtedly affect lower or higher. The selection of articles in English may be another limitation of this study.

## Conclusions

In this meta-analysis and systematic review of 11 separate investigations, high rates of depression, anxiety, distress, anxiety, dread, and other uncommon symptoms were observed in a wide range of research designs. Clinicians must consider the clinical implications of developing COVID-19 symptoms in patients, which may include depression, anxiety, exhaustion, post-traumatic stress disorder, and a very small percentage of cases of COVID-19 infection, as well as rare prevalence and event rates need to be considered by clinicians.

### Supplementary Information

**Additional file 1. **

## Data Availability

All data generated or analysed during this study are included in this published article within the figures, tables and supplementary material.
